# Impact of preoperative transcatheter arterial chemoembolization (TACE) on postoperative long-term survival in patients with nonsmall hepatocellular carcinoma: a propensity score matching analysis

**DOI:** 10.1186/s12885-024-11978-4

**Published:** 2024-02-09

**Authors:** Run Hu, Jie Xu, Hongxiang Wang, Jiaguo Wang, Kai Lei, Xiaoping Zhao, Huizhi Zhang, Ke You, Zuojin Liu

**Affiliations:** The Second Affiliated Hospital of Chongqing Medical University, 74# Linjiang Road, Yuzhong District, Chongqing, 400000 China

**Keywords:** Nonsmall hepatocellular carcinoma, Hepatectomy, Transcatheter arterial chemoembolization, Progression-free survival, Overall survival, Propensity score matching

## Abstract

**Background:**

The purpose of this propensity score matching (PSM) analysis was to compare the effects of preoperative transcatheter arterial chemoembolization (TACE) and non-TACE on the long-term survival of patients who undergo radical hepatectomy.

**Methods:**

PSM analysis was performed for 387 patients with hepatocellular carcinoma (HCC) (single > 3 cm or multiple) who underwent radical resection of HCC at our centre from January 2011 to June 2018. The patients were allocated to a preoperative TACE group (*n* = 77) and a non-TACE group (*n* = 310). The main outcome measures were progression-free survival (PFS) and overall survival (OS) since the treatment date.

**Results:**

After PSM, 67 patients were included in each of the TACE and non-TACE groups. The median PFS times in the preoperative TACE and non-TACE groups were 24.0 and 11.3 months, respectively (*p* = 0.0117). The median OS times in the preoperative TACE and non-TACE groups were 41.5 and 29.0 months, respectively (*p* = 0.0114). Multivariate Cox proportional hazard regression analysis revealed that preoperative TACE (hazard ratio, 1.733; 95% CI, 1.168–2.570) and tumour thrombosis (hazard ratio, 0.323; 95% CI, 0.141–0.742) were independent risk factors significantly associated with OS.

**Conclusions:**

Preoperative TACE is related to improving PFS and OS after resection of HCC. Preoperative TACE and tumour thrombus volume were also found to be independent risk factors associated with OS.

## Introduction

Primary liver cancer, which consists predominantly of hepatocellular carcinoma (HCC), is the fifth most common cancer worldwide and the third most common cause of cancer mortality. HCC accounts for between 85% and 90% of primary liver cancers [1]. Generally, surgical resection is a good choice for patients with early or intermediate HCC. However, for small liver cancer, which is defined as a HCC involving one to three tumours with a diameter of 3 cm or less [2], it has been reported that the therapeutic effect of radiofrequency ablation (RFA) is not inferior to that of surgical resection. In some studies, RFA and hepatectomy can be used as first-line treatments for isolated HCCs smaller than 3 cm. It is believed that RFA can achieve the same effect as hepatectomy or even have fewer complications [3–6]. Surgical resection is the primary method for treating hepatocellular carcinoma larger than 3 cm for curative purposes [7]. However, even in patients with early disease, approximately 50% and 70% of patients will experience recurrence and metastasis after surgery, respectively [7, 8]. Hence, measures aimed at mitigating tumour recurrence and extending the postoperative survival period are urgently needed.

According to the Barcelona Clinical Hepatocellular Carcinoma (BCLC) staging system, TACE is the first choice for the treatment of midterm HCC, which includes unresectable and unresectable multinodular HCC without extrahepatic spread [9]. Several studies have evaluated the effectiveness of preoperative TACE for preventing recurrence and prolonging survival after resection of liver cancer [10–17]. However, the conclusions of these studies are controversial. Therefore, subsequent studies by some scholars have largely failed to provide evidence supporting the survival benefits of conventional preoperative TACE in all patients who undergo hepatectomy [10–19]. However, several authors suggest that TACE may benefit certain types of liver cancer patients [20–26]. 

Therefore, this study was designed to investigate the impact of preoperative TACE on PFS and OS in patients with nonsmall hepatocellular carcinoma. To clarify the association between preoperative TACE and postoperative tumour prognosis, PSM analysis was employed to balance differences in baseline characteristics between the two groups.

## Materials and methods

### Patients

This study was approved by the Ethics Committee of the Second Affiliated Hospital of Chongqing Medical University. Before receiving any treatment, all patients were informed of the risks and provided written informed consent. To analyse the impact of preoperative TACE on the long-term survival of patients with liver cancer, an electronic medical record platform was used to collect data from patients with nonsmall hepatocellular carcinoma who underwent continuous R0 resection at our hospital from January 2011 to June 2018. Preoperative diagnosis of HCC was based on the diagnostic criteria used by the American Association for the Study of Liver Diseases [27]. The patients included in the analysis cohort had (a) a maximum diameter of a single tumour > 3 cm or multiple tumours, and the resected specimens were confirmed by histopathological examination; (b) no distant metastasis; and (c) radical hepatectomy, that is, complete resection of all microscopic and naked eye tumours (R0 resection) [28]. The following patients were excluded: (a) patients who had liver cancer under 18 years of age; (b) patients who had a maximum diameter of a single tumour ≤ 3 cm; (c) patients who had recurrent liver cancer; (d) patients who received preoperative anti-liver cancer therapy other than TACE, including portal vein embolization, systematic chemotherapy or RFA; (e) patients who had palliative hepatectomy, that is, microscopic positive (R1 resection) or gross positive (R2 resection); (f) patients who were lost to follow-up within 90 days after hepatectomy; and (g) patients who lacked data on prognostic variables or follow-up information.

### Preoperative TACE

The Seldinger technique was used for percutaneous puncture of the right femoral artery, and a short guide wire was used to insert a 5-Fr micro needle catheter sleeve (RCFN; COOK). Then, under X-ray fluoroscopy, a 5-Fr angiography catheter (HNBR; COOK) was inserted into the abdominal trunk and common hepatic artery for angiography to visualize the distribution of feeding arteries and tumour vessels. A 2.7-Fr angiographic microcatheter (MF; Cook) was superselectively inserted into the tumour nutrient artery through the microcatheter, and chemotherapeutic drugs were injected into the tumour nutrient artery through the microcatheter. The specific regimens used were pirarubicin (20 mg) and lipiodol (5–20 mL). The actual dose was determined according to the size and number of tumours and the liver function of the patients [29]. After embolization with a lipiodol emulsion, a blank microsphere embolization agent was used to determine the diameter of the microspheres according to the tumour size and blood supply [30]. Five minutes after injection, the blood flow of the tumour trophoblast artery was confirmed by digital subtraction angiography (DSA).

### Baseline characteristics and follow-up

Routine preoperative examination included imaging and serological examination. All patients underwent abdominal ultrasound, enhanced abdominal MRI and/or CT, and chest CT. All radiological data were reviewed by two independent radiologists with more than 10 years of radiology experience using uniform diagnostic criteria. Information about the baseline characteristics of the patients included age, sex, background of liver disease, Child‒Pugh grade, BCLC stage, prognostic nutritional index (PNI) score, preoperative albumin level, preoperative alpha-fetoprotein (AFP) level, preoperative alanine aminotransferase (ALT) level, preoperative aspartate aminotransferase (AST) level, preoperative total bilirubin (TBIL) level, preoperative alkaline phosphatase (ALP) level, tumour number, tumour maximum size, total tumour size, tumour differentiation, and tumour thrombosis.

Patients were followed up regularly in our hospital. Postoperative recurrence was monitored by AFP levels, ultrasound, or contrast-enhanced CT/magnetic resonance imaging (MRI) of the abdomen every 1 month for the first 6 months after resection, every 3 months for the following 18 months, and every 6 months thereafter. When HCC recurrence was suspected, CT and/or MRI was performed as clinically indicated. Tumour recurrence was defined as the emergence of new intrahepatic or extrahepatic nodules with or without elevated AFP levels, and intrahepatic nodules had typical imaging features consistent with HCC on enhanced CT or MRI. Treatment options for patients with recurrent tumours included TACE, reoperation, liver transplantation, RFA, targeted therapy, and immunotherapy.

### Study endpoints

The endpoints of the study included OS and PFS. OS was defined as the time from surgery to death from any cause, while PFS was defined as the time from the date of operation to the date when a patient with recurrence was first diagnosed with recurrent liver cancer or from the date of operation to the date of the last follow-up or death in patients without recurrence.

### Propensity score matching (PSM)

To reduce potential bias caused by covariates, PSM analysis with logistic regression was performed [31]. The covariates entered into the PSM model included age, sex, background of liver disease, Child‒Pugh grade, BCLC stage, PNI score, serum ALB concentration, AFP, ALT, AST, TBIL, ALP, tumour number, tumour maximum size, total tumour size, tumour differentiation, and portal vein tumour thrombosis. The two groups were matched at a 1:1 ratio, the nearest neighbour was matched, and the calliper width was 0.02 mm.

### Statistical analysis

All the statistical analyses were carried out with SPSS 26.0 for Windows (SPSS, Inc., Armonk, NY, USA) and GraphPad Prism version 8.0.2 for Windows (GraphPad, Inc., San Diego, California, USA). *p* values < 0.05 were considered to indicate statistical significance. Continuous variables are expressed as the mean ± standard deviation (SD). Categorical variables are reported as numbers (N) or proportions (%). Student’s t test was used for comparisons of continuous variables when applicable. Otherwise, the Mann‒Whitney U test was applied. Categorical variables were compared with the χ2 test with the Yates correction or Fisher’s exact test as appropriate. PFS and OS were compared among patients who did and did not undergo preoperative TACE before and after PSM using Kaplan–Meier curves generated by the log-rank test. Univariate and multivariate Cox proportional hazard regression analyses were subsequently performed to identify other prognostic factors that were associated with PFS and OS.

## Results

**Fig. 1:**
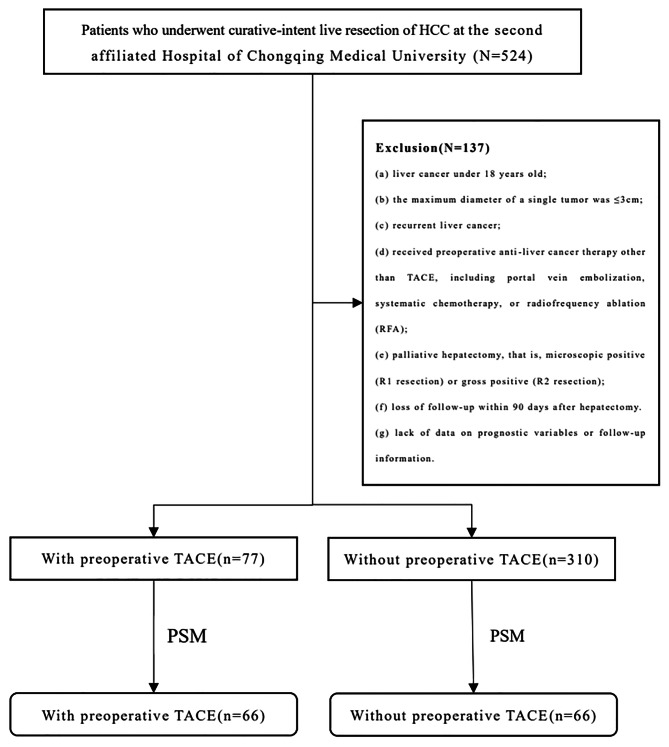
Selection of the study population. HCC: hepatocellular carcinoma; TACE: transcatheter arterial chemoembolization; PSM: propensity score matching

During the study period, 524 HCC patients underwent hepatectomy, 387 of whom met the inclusion criteria; these patients composed the analysis cohort (Fig. 1). Among these 387 patients, 77 patients underwent preoperative TACE at least once before hepatectomy. PSM was used to identify 67 pairs of patients who did or did not undergo preoperative TACE.

### Patient characteristics

The baseline data at the time of initial diagnosis in the preoperative TACE group were compared with the baseline data in the group without TACE before and after PSM as shown in Table 1. A total of 387 patients who underwent radical hepatectomy were included in this study; 77 patients underwent preoperative TACE, and 310 patients did not. Before the operation, there were 63 males (81.8%) and 14 females (18.2%) in the preoperative TACE group, with a mean age of 51.7 ± 11.26 years. There were 275 males (88.7%) and 35 females (11.3%) in the nonpreoperative TACE group, with a mean age of 52.63 ± 11.50 years. There were no significant differences in age, sex, background of liver disease, Child‒Pugh grade, BCLC stage, AFP, longest diameter, overall tumour size, or tumour thrombosis between the two groups. The tumour number in patients who underwent preoperative TACE was significantly lower than that in patients who did not. PSM analysis produced 72 pairs of patients. The baseline characteristics of patients in the two groups in the tendency-matching cohort were compared as shown in Table 1. After PSM, there was no significant difference in background characteristics or preoperative factors between the two groups.

**Table 1:** Baseline data at the time of initial diagnosis in the preoperative transcatheter arterial chemoembolization(TACE) group were compared with baseline data in the group without TACE before and after propensity score matching (PSM)

N(%)	The entire cohort					The PSM cohort			
	With Preoperative		Without Preoperative			With Preoperative		Without Preoperative	
	TACE group*		TACE group			TACE group*		TACE group	
	(*N* = 77)		(*N* = 310)			(*N* = 72)		(*N* = 72)	
Variables	N (%)		N (%)	p		N (%)		N (%)	p
Age, years (Mean ± SD)	51.7 ± 11.26		52.63 ± 11.50	0.525		51.89 ± 11.36		50.70 ± 9.85	0.521
Gender									
Male	63(81.8)		275(88.7)	0.104		58(80.6)		57(79.2)	0.835
Female	14(18.2)		35(11.3)			14(19.4)		15(20.8)	
Background liver disease									
-	0(0)		5(1.6)	0.366		0(0)		0(0)	0.354
HBV	67(87.0)		253(81.6)			63(87.5)		59(81.9)	
HCV	10(13.0)		52(16.8)			9(12.5)		13(18.1)	
C-P classification									
A	77(100)		308(99.4)	0.48		71(100)		71(100)	1
B	0(0)		2(0.6)			0(0)		0(0)	
BCLC stage									
A	62(80.5)		263(84.8)	0.432		58(80.6)		54(75.0)	0.718
B	10(13.0)		28(10.1)			9(12.5)		12(16.7)	
C	5(6.5)		19(6.1)			5(6.9)		6(8.3)	
AFP(ug/L)	38.3(4.93–306.5)		50.7(6.76-757.85)	0.107		35.32(5.18-302.63)		40.90(7.12-334.75)	0.436
Tumor number									
Single	56(72.7)		261(84.2)	**0.019**		53(73.6)		51(70.8)	0.71
Multiple	21(27.3)		49(15.8)			19(26.4)		21(29.2)	
Tumor longest diameter(mm)	34.0(23.2–56.3)		42.96(25-64.08)	0.64		35.11(30.00-56.75)		45.00(26.00–70.00)	0.448
Tumor overall size(mm)	35.1(30.0-57.9)		45(27-67.7)	0.979		37.05(31.00-57.68)		47.26(29.23–74.25)	0.431
Tumor thrombus									
+	7(9.0)		19(6.1)	0.353		4(5.6)		4(5.6)	1
-	70(91.0)		291(93.9)			68(94.4)		68(94.4)	
Tumor response									
CR	6(7.8)			-		5(6.9)			-
PR	30(39.0)					30(38.9)			
SD	28(36.4)					27(37.5)			
PD	13(16.9)					20(16.7)			

Abbreviations: TACE: transcatheter arterial chemoembolization; HBV: hepatitis B virus; HCV: hepatitis C virus; C-P Grade: Child-Pugh Grade; BCLC: Barcelona Clinic Liver Cancer; AFP: serum alpha-fetoprotein; CR: complete response; PR: partial response; SD: stable disease; PD: progressive disease

* Baseline data for the initial diagnosis in the preoperative TACE group

We also compared baseline data before hepatectomy between the groups of patients with and without preoperative TACE in Table 2. A total of 387 patients who underwent radical hepatectomy were included in this study; 77 patients underwent preoperative TACE, and 310 patients did not. Before the operation, there were 63 males (81.8%) and 14 females (18.2%) in the preoperative TACE group, with a mean age of 51.7 ± 11.26 years. There were 275 males (88.7%) and 35 females (11.3%) in the nonpreoperative TACE group, with a mean age of 52.63 ± 11.50 years. There were no significant differences in age, sex, background of liver disease, Child‒Pugh grade, BCLC stage, serum ALB concentration, total bilirubin (TBIL) level, number of tumours, tumour thrombosis, or tumour differentiation between the two groups. However, the preoperative ALT, AST, ALP, PNI score, tumour longest diameter, and overall tumour size were significantly lower in patients who underwent preoperative TACE than in those who did not. PSM analysis resulted in 67 pairs of patients. The baseline characteristics of the two groups of patients in the tendency-matching cohort were compared as shown in Table 2. After PSM, there was no significant difference in background characteristics or preoperative factors between the two groups.

**Table 2:** Comparisons of patients’ baseline characteristics before hepatectomy between with and without preoperative TACE group before and after PSM

N(%)	The entire cohort					The PSM cohort			
	With Preoperative		Without Preoperative			With Preoperative		Without Preoperative	
	TACE Group**		TACE Group			TACE Group		TACE Group	
	(*N* = 77)		(*N* = 310)			(*N* = 67)		(*N* = 67)	
Variables	N (%)		N (%)	p		N (%)		N (%)	p
Age, years (Mean ± SD)	51.7 ± 11.26		52.63 ± 11.50	0.525		52 ± 11.47		50 ± 11.171	0.632
Gender									
Male	63(81.8)		275(88.7)	0.525		56(83.6)		55(82.1)	0.819
Female	14(18.2)		35(11.3)			11(16.4)		12(17.9)	
Background liver disease									
-	0(0)		5(1.6)	0.366		0(0)		1(1.5)	0.456
HBV	67(87.0)		253(81.6)			58(86.6)		54(80.6)	
HCV	10(13.0)		52(16.8)			9(13.4)		12(17.9)	
C-P Grade									
A	77(100)		308(99.4)	0.480		67(100)		67(100)	1
B	0(0)		2(0.6)			0(0)		0(0)	
BCLC stage									
A	61(79.2)		263(84.8)	0.128		56(83.6)		57(85)	0.548
B	11(14.3)		28(10.1)			8(11.9)		5(7.5)	
C	5(66.4)		19(6.1)			3(4.5)		5(7.5)	
ALB (g/L)	40.9(37.2-43.65)		41.2(38.1-44.35)	0.276		41(38.1–43.8)		40.6(37.8–44.8)	0.546
AFP(ug/L)	38.3(4.93–312.9)		50.7(6.76-757.85)	0.137		26.16(4.93–291)		20.62(5.61–222.9)	0.895
ALT(U/L)	29(21–47)		38(26–57)	**0.012**		28(21–48)		36(23–52)	0.110
AST(U/L)	31(24–47)		36(28–55)	**0.088**		29(24–45)		35(28–52)	0.104
TBIL(umol/L)	12.9(8.8–19.6)		13.75(9.8-19.15)	0.254		12.5(8.5–20.4)		12.5(8.9–20.7)	0.431
ALP(U/L)	84(70–109)		93.5(75–122)	**0.022**		84(71–110)		85(72–116)	0.677
PNI score	46.25(43-50.075)		48(44.69–51.21)	**0.030**		46.25(43.35–50.5)		46.9(44.8-50.85)	0.457
Tumor number									
Single	59(76.6)		261(84.2)	0.116		55(82.1)		55(82.1)	1
Multiple	18(23.4)		49(15.8)			12(17.9)		12(17.9)	
Tumor longest diameter(mm)	30.36(17.5–55.5)		42.96(25-64.08)	**0.006**		30.36(17–54)		40(22–58)	0.149
Tumor overall size	33(21.2–56.5)		45(27-67.7)	**0.013**		31(22–54)		41(27–61)	0.098
Tumor thrombus									
+	5(20.8)		19(6.1)	0.906		3(4.5)		5(7.5)	0.466
-	72(19.8)		291(93.9)			64(95.5)		62(92.5)	
Degrees of differentiation									
Well or moderate	73(94.8)		280(90.3)	0.209		64(95.5)		61(91.1)	0.819
Poor	4(5.2)		30(9.7)			3(4.5)		6(8.9)	

Abbreviations: TACE: transcatheter arterial chemoembolization; HBV: hepatitis B virus; HCV: hepatitis C virus; C-P Grade: Child-Pugh Grad; BCLC: Barcelona Clinic Liver Cancer; ALB: albumin; AFP: serum alpha-fetoprotein; ALT: alanine aminotransferase; AST: aspartate aminotransferase; TBIL: total bilirubin; PNI score: Prognostic Nutritional Index score; PSM: Propensity score matching

** Baseline data of the preoperative TACE group before hepatectomy

### Progression-free survival (PFS) and overall survival (OS) between patients with and without preoperative TACE

**Fig. 2:**
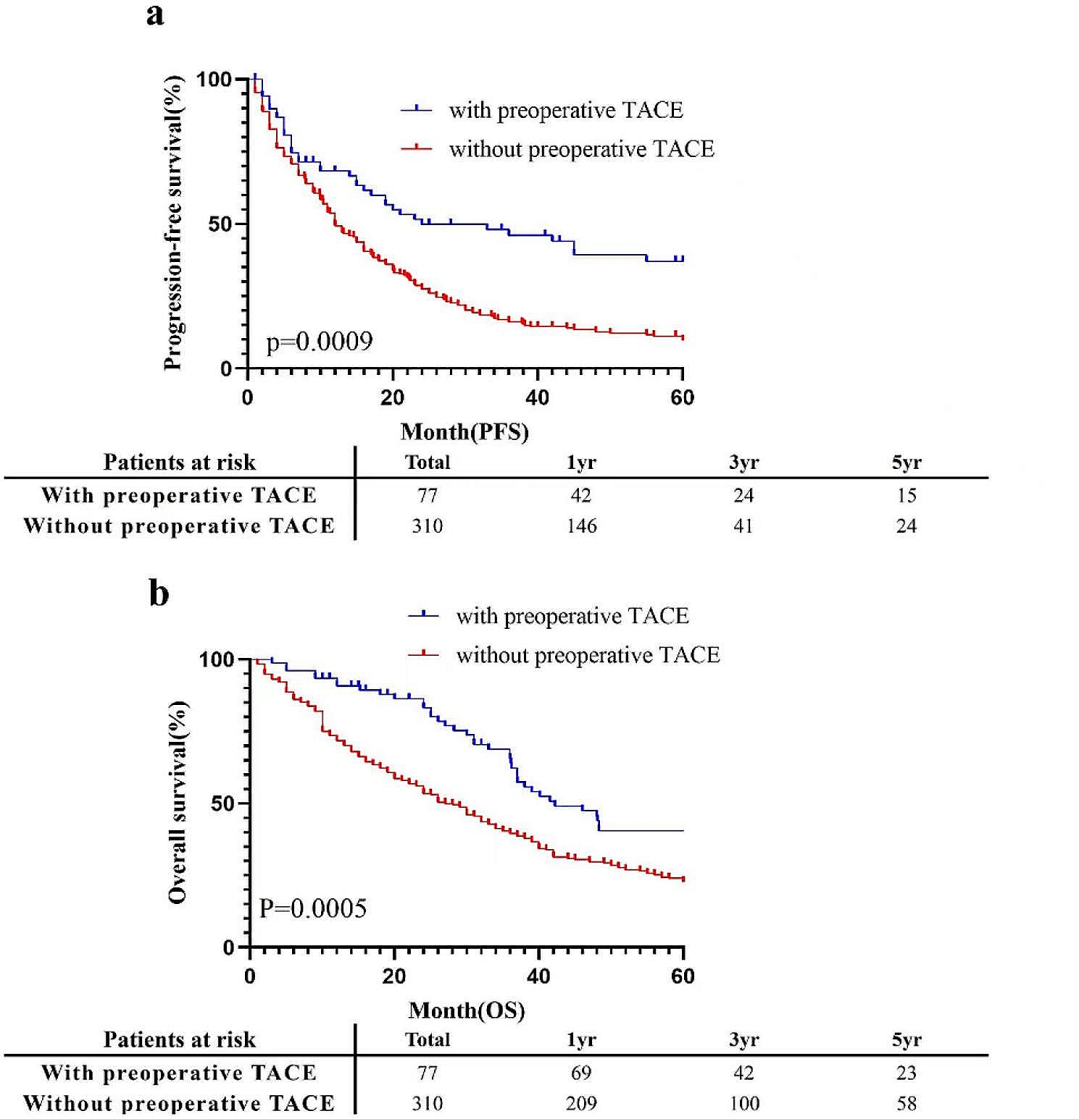
Cumulative incidence of PFS (**a**) and OS (**b**) curves comparisons between patients with and without preoperative TACE in the entire cohort. TACE: transcatheter arterial chemoembolization; PFS: progression-free survival; OS: overall survival

Among the patients who participated in the study, the OS and PFS of patients who received TACE before the operation and those who did not receive TACE are shown in Fig. 2. In the whole cohort analysis, the median OS and PFS of patients who received TACE were 42.2 and 24.0 months, respectively, and the median OS and PFS of patients without TACE were 27.0 and 12.0 months, respectively. The OS of patients who received preoperative TACE was significantly greater than that of patients who did not receive TACE (*p* = 0.0005), and there was a significant difference in PFS between patients who received preoperative TACE and those who did not receive preoperative TACE (*p* = 0.0009).

**Fig. 3:**
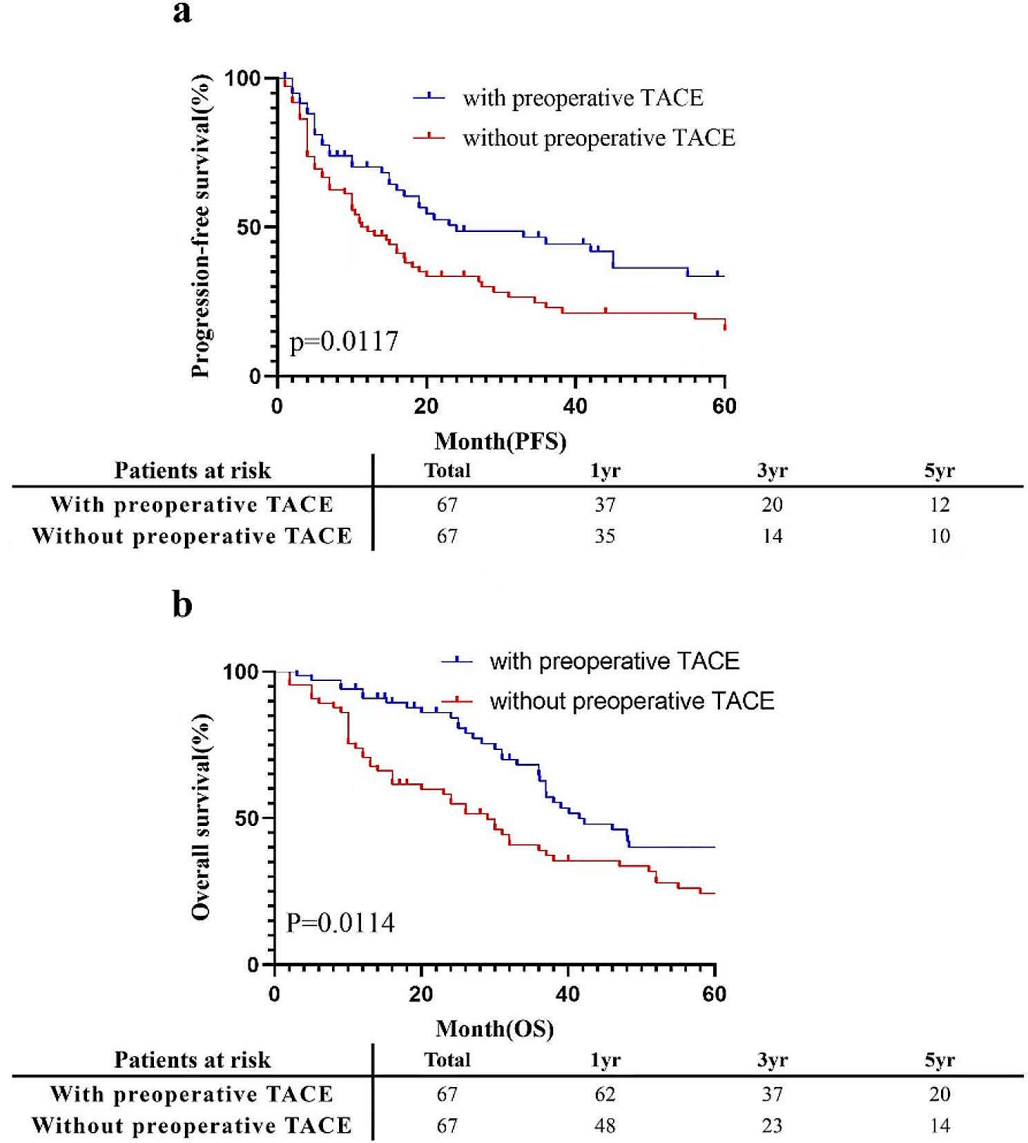
Cumulative incidence of PFS (**a**) and OS (**b**) curves comparisons between patients with and without preoperative TACE in the PSM cohort. TACE: transcatheter arterial chemoembolization; PFS: progression-free survival; OS: overall survival; PSM: propensity score-matching

After PSM, the median OS and PFS of patients who received preoperative TACE were 41.5 and 24.0 months, respectively, and those of patients who did not receive preoperative TACE were 29.0 and 11.3 months, respectively. (Fig. 3) The OS of patients who received preoperative TACE was also better than that of patients who did not receive TACE (*p* = 0.0114), and the difference in PFS between patients who received preoperative TACE and those who did not receive TACE was also significant (*p* = 0.0117). The 1-, 3- and 5-year OS rates with preoperative TACE were 92.5%, 55.2%, and 26.7%, respectively, while those without preoperative TACE were 71.6%, 34.3%, and 20.9%, respectively (*p* = 0.0114). The 1-, 3- and 5-year PFS rates with preoperative TACE were 55.2%, 29.9%, and 17.9%, respectively, while those without preoperative TACE were 55.2%, 20.9%, and 14.9%, respectively (*p* = 0.0117).

**Fig. 4:**
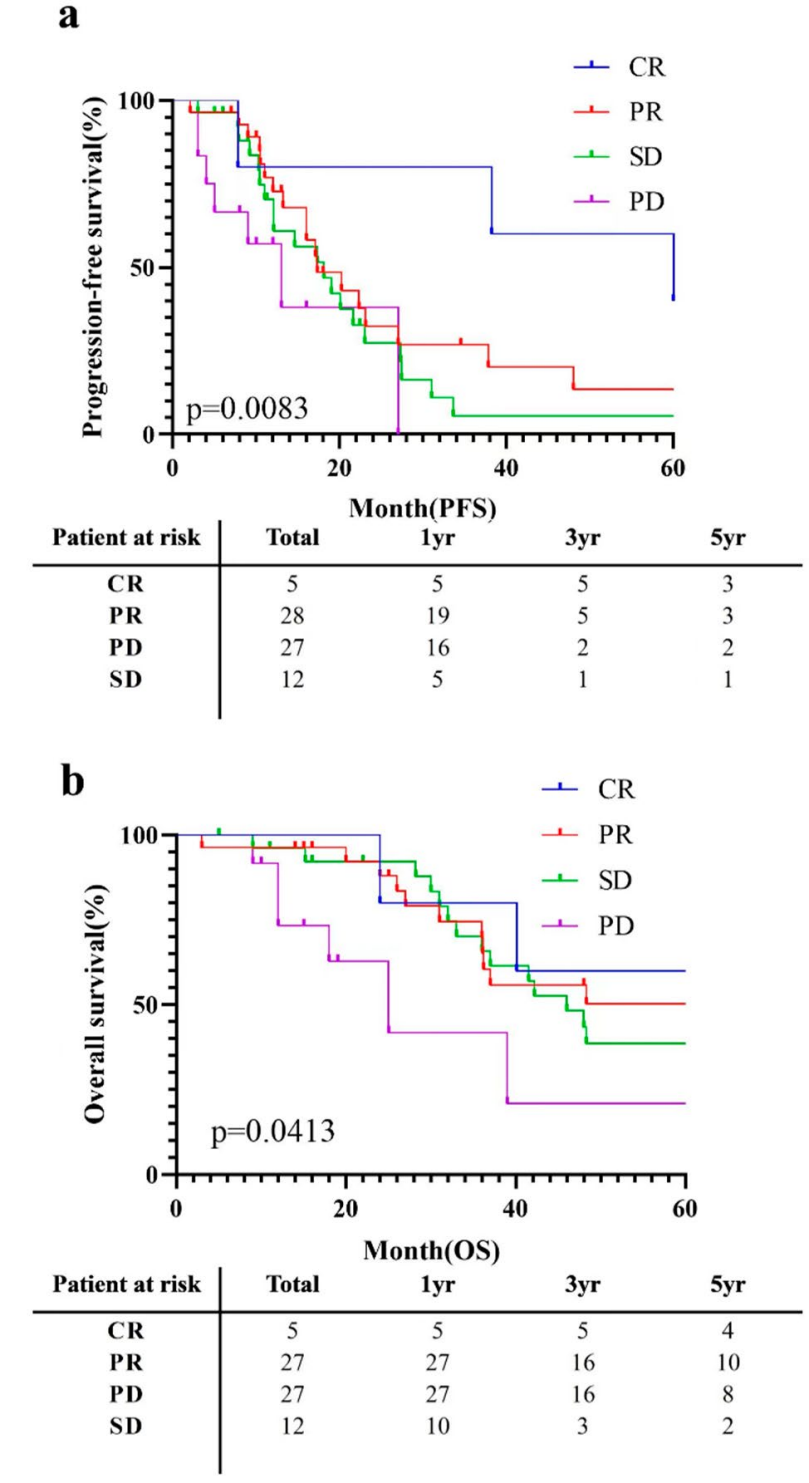
Comparison of cumulative PFS (**a**) and OS (**b**) curves among preoperative TACE patients in the PSM cohort among different tumour response subgroups. CR: complete response; PR: partial response; SD: stable disease; PD: progressive disease; PFS: progression-free survival; OS: overall survival; TACE: transcatheter arterial chemoembolization; PSM: propensity score-matching

Moreover, subgroup analysis of survival curves was performed for patients with different degrees of tumour response in the preoperative TACE group. (Fig. 4) The median OS was 91.0 months for complete response (CR), 60.4 months for partial response (PR), 46.0 months for stable disease (SD), and 25.0 months for progressive disease (PD) (*p* = 0.0413). In addition, the median PFS was 60.0 months for CR, 17.3 months for PR, 18.1 months for SD, and 13.0 months for PD (*p* = 0.0083).

### Univariable and multivariable analyses of OS and PFS in the PSM cohort

Univariate and multivariate Cox regression analyses with robust estimates were also carried out for the PSM cohort. Univariate and multivariate analyses of PFS and OS after radical hepatectomy for HCC in the PSM cohort are shown in Tables 3 and 4. Univariate analysis also revealed that preoperative TACE was correlated with OS. According to our multivariate analysis, preoperative TACE was still independently correlated with survival. According to the univariate analysis, there was no correlation between preoperative TACE and PFS. According to our multivariate analysis, BCLC stage and AFP were correlated with PFS.

**Table 3:** Univariate and multivariate Cox-regression analyses predicting progression-free survival in the PSM cohort

Variables	Univariate			Multivariate	
	HR (95% CI)	*p*		HR (95% CI)	*p*
Preoperative TACE(No vs. yes)	0.772(0.325–1.833)	0.557		0.841(0.193–3.761)	0.818
Gender (male vs. female)	0.811(0.335–1.966)	0.643		0.696(0.36.-2.054)	0.512
Age	0.999(0.966–1.033)	0.956		0.975(0.02.-1.016)	0.229
Background liver disease(No vs. yes)	0.622(0.083–4.644)	0.643		0.583(0.54.-6.401)	0.659
BCLC stage (A vs. B or C)	0.227(0.031–1.686)	0.147		0.066(2.71.-0.814)	**0.034**
C-P grade(A vs. B or C)	0.048(0.000-12739.032)	0.633		-	--
ALB(g/L)	1.017(0.964–1.074)	0.534		1.185(0.17.-1.511)	0.170
AFP(ug/L)	0.999(0.997-1.000)	0.110		0.998(0.00.-1.000)	**0.039**
ALT(U/L)	0.991(0.977–1.005)	0.193		0.988(0.01.-1.005)	0.173
AST(U/L)	0.988(0.970–1.005)	0.163		1.004(0.00.-1.029)	0.769
TBIL(umol/L)	0.991(0.956–1.028)	0.628		1.003(0.00.-1.016)	0.706
ALP(U/L)	0.998(0.987–1.009)	0.662		1.034(0.03.-1.114)	0.376
PNI score	1.004(0.948–1.062)	0.904		0.846(0.16.-1.089)	0.194
Tumor number(Solitary vs. multiple)	1.464(0.543–3.947)	0.452		2.620(0.96.-22.463)	0.380
Tumor longest diameter	0.996(0.985–1.006)	0.395		0.957(0.04.-1.141)	0.627
Tumor overall size	0.996(0.986–1.006)	0.418		1.034(0.03.-1.231)	0.707
Tumor thrombus(No vs. yes)	2.268(0.303–16.967)	0.425		0.630(0.46.-23.247)	0.802
Degrees of differentiation(Well or moderate vs. Poor)	0.598(0.076–4.688)	0.625		1.094(0.09.-10.277)	0.938
Times	1.076(0.823–1.405)	0.592		0.992(0.00.-1.709)	0.976

Abbreviations: TACE: transcatheter arterial chemoembolization; BCLC: Barcelona Clinic Liver Cancer; C-P grade: Child-Pugh grade; ALB: albumin; AFP: alpha-fetoprotein; ALT: alanine aminotransferase; AST: aspartate aminotransferase; TBIL: total bilirubin; PNI score: Prognostic Nutritional Index score; HR: hazard ratio; 95% CI: 95% confidence interval; PSM: Propensity score matching

**Table 4:** Univariate and multivariate Cox-regression analyses predicting overall survival in the propensity score-matching (PSM) cohort

Variables	Univariate			Multivariate	
	HR (95% CI)	*p*		HR (95% CI)	*p*
Preoperative TACE(No vs. yes)	1.639(1.111–2.420)	**0.013**		1.733(1.168–2.570)	**0.006**
Gender (male vs. female)	1.756(0.994–3.101)	**0.040**		1.651(0.916–2.976)	0.095
Age	1.020(1.001–1.038)	**0.034**		1.018(0.998–1.037)	0.073
Background liver disease(No vs. yes)	1.219(0.950–1.563)	0.119			
BCLC stage (A vs. B or C)	0.273(0.037–2.022)	0.659			
Child-Pugh grade(A vs. B or C)	-	-			
ALB(g/L)	0.960(0.924–0.996)	**0.030**		0.969(0.930–1.009)	0.126
AFP(ug/L)	1.000(1.000-1.001)	0.685			
ALT(U/L)	1.001(0.999–1.004)	0.313			
AST(U/L)	1.003(1.001–1.006)	**0.022**		1.003(1.000-1.005)	0.061
TBIL(umol/L)	0.994(0.973–1.015)	0.589			
ALP(U/L)	1.005(1.001–1.009)	**0.032**			
PNI score	0.978(0.946–1.011)	0.182			
Tumor number(Solitary vs. multiple)	1.006(0.602–1.681)	0.981			
Tumor longest diameter	1.000(0.998–1.002)	0.975			
Tumor overall size	1.000(0.998–1.002)	0.984			
Tumor thrombus(No vs. yes)	0.251(0.112–0.561)	**0.001**		0.323(0.141–0.742)	**0.008**
Degrees of differentiation(Well or moderate vs. Poor)	0.507(0.246–1.048)	0.094			
Times	0.834(0.707–0.987)	**0.026**		0.962(0.743–1.245)	0.768

Abbreviations: TACE: transcatheter arterial chemoembolization; BCLC: Barcelona Clinic Liver Cancer; C-P grade: Child-Pugh grade; ALB: albumin; AFP: alpha-fetoprotein; ALT: alanine aminotransferase; AST: aspartate aminotransferase; TBIL: total bilirubin; PNI score: Prognostic Nutritional Index score; HR: hazard ratio; 95% CI: 95% confidence interval; PSM: Propensity score matching

### Subgroup analysis after PSM

**Fig. 5:**
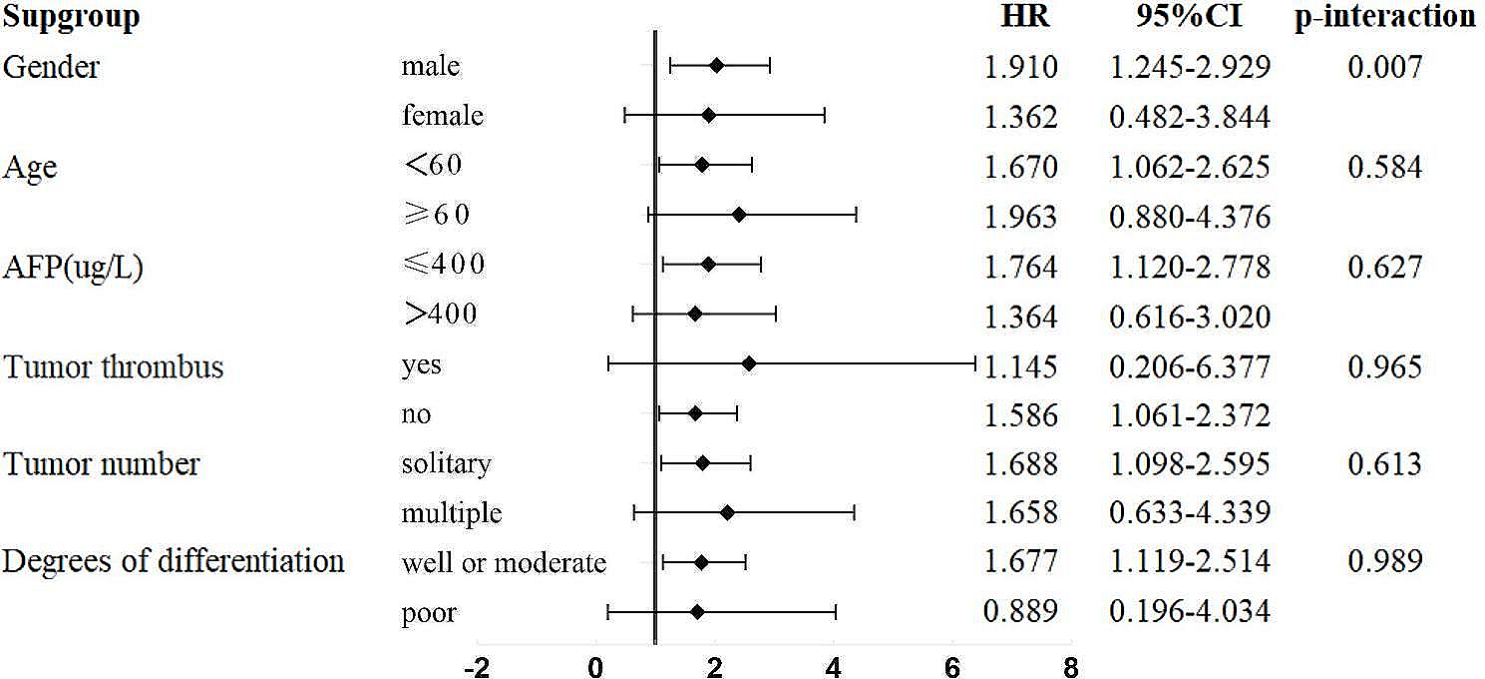
Subgroup analysis: progression hazard ratios according to baseline covariates. P-interaction was calculated from a likelihood ratio test. HR: hazard ratios; CI: confidence interval; AFP: alpha fetoprotein

To further determine the potential factors affecting the preoperative effect of TACE on the prognosis of patients who underwent radical hepatectomy, a subgroup analysis was performed after PSM (Fig. 5). Potential confounding variables included sex, age, AFP concentration, tumour number, tumour thrombosis, and degree of differentiation. Although the total sample size of patients with nonsmall HCC was more than 300 (including 77 patients who underwent preoperative TACE), the sample size was still relatively small. According to our subgroup analysis, among the groups with large sample sizes, there was a significant correlation between preoperative TACE and OS (*p* < 0.05), but there was no significant difference in predictive ability (all P-interaction > 0.05).

## Discussion

Given the persistently high recurrence rate of 70–80%, even after curative liver resection (R0 LR), which significantly impairs the long-term survival of HCC patients [32, 33], we are actively seeking to identify factors that can effectively mitigate postoperative recurrence in this population. Due to its ability to induce tumour necrosis and shrinkage, TACE has garnered considerable attention as a standard locoregional life-extending treatment for unresectable HCC [34]. Nevertheless, the efficacy of preoperative TACE in radical resection of hepatocellular carcinoma remains controversial. In several studies, scholars have reported that a subset of resectable HCC patients may benefit from preoperative TACE [20–26]. Therefore, we chose a subset of patients whom we thought might benefit. For the selection of patients, as shown in the above flow chart (Fig. 1), we selected liver cancer patients with a single lesion larger than 3 cm or multiple lesions, which indicated that these patients could have a high risk of postoperative recurrence. Therefore, we were considering whether preoperative TACE could improve the postoperative survival outcomes of these patients. Thus, this study focused on the long-term prognosis of patients with nonsmall HCC (single > 3 cm or multiple) who underwent radical hepatectomy. According to the evaluation of the whole cohort and the PSM cohort, preoperative TACE significantly improved the PFS and OS of patients. In addition, multivariate Cox regression analysis showed that preoperative TACE and tumour thrombus volume were still strongly correlated with OS in patients who underwent radical hepatectomy. Moreover, in preoperative TACE patients, the survival prognosis was greatly improved in patients with a better tumour response.

Most baseline characteristics did not differ among patients who did and did not undergo preoperative TACE except for ALT, AST, ALP, and PNI score or tumour longest diameter. The imbalance in these baseline characteristics is likely related to the inherent selection bias caused by the retrospective nature of the study. Patients with poor liver function and larger tumours were more likely to receive preoperative TACE.

To reduce the potential bias caused by covariates, we used the PSM method to adjust for potential confounding factors and reduce selection bias between the two groups. After PSM, there was no significant difference in background characteristics or preoperative factors between the two groups.

For long-term outcomes, the results demonstrated that long-term PFS and OS after curative resection of HCC were better in patients who had preoperative TACE than in patients who did not have preoperative TACE (median PFS and OS in the entire cohort: 24.0 and 42.2 months vs. 11.3 and 27.0 months, *p* = 0.0009 and *p* = 0.0005; median PFS and OS in the PSM cohort: 24.0 and 41.5 months vs. 11.3 and 29.0 months, *p* = 0.0117 and *p* = 0.0114, respectively). Due to the high risk of HCC recurrence, all patients received a multidisciplinary consultation after surgery, and all patients were treated with targeted immunization and interventional therapy according to their conditions. In our study, patients in the preoperative TACE group had better PFS and OS than did those in the nonpreoperative TACE group. Based on the data from the above retrospective study, we believe that this subgroup of patients may benefit from preoperative TACE.

The effect of preoperative TACE in this study can be attributed to several factors. First, in terms of molecular mechanisms, preoperative TACE regimens may enhance the apoptosis of HCC cells by upregulating the expression of the Bax protein and downregulating the expression of the Bcl-2 protein and the ratio of Bcl-2 to Bax protein expression [35]. Second, TACE enhances the expression of the metastasis suppressor genes *nm23-H1* and *TIMP-2*, possibly inhibiting the metastasis of hepatocellular carcinoma [36]. Third, the proportion of regulatory T (Treg) cells in the peripheral blood of patients with hepatocellular carcinoma can be significantly reduced by microparticle-TACE (MTACE), indicating that mTACE has a positive regulatory effect on anticancer immune function in patients with hepatocellular carcinoma [37]. Furthermore, preoperative TACE potentially improves long-term progression after resection by reducing the percentage of MVI-positive patients [20–26]. MVI in hepatocellular carcinoma is common in HCC and is related to early tumour recurrence and reduced survival outcomes [38]. Therefore, preoperative TACE can promote tumour reduction to a certain extent and transform unresectable liver cancer into resectable liver cancer; this not only expands the indication for radical resection of HCC but also reduces the possibility of tumour metastasis [21, 39, 40]. In addition, preoperative TACE can promote the formation of tumour capsules and increase capsule thickness to reduce the rate of tumour metastasis and increase the rate of RO resection [25]. This may be further supported by the finding that PFS and OS in patients who achieved CR and PR with preoperative TACE were better than those in patients with PD and SD. Moreover, preoperative TACE can reveal minor lesions with a diameter smaller than 2 mm [41], helping to prevent incomplete resection by surgeons during the process of resection and thus reducing the risk of early recurrence.

Due to the limitation of sample size, the results of the subgroup analysis were not satisfactory. However, these findings also indicate that preoperative TACE is beneficial for patient survival.

There are several limitations to our study. First, this was a retrospective study that lacked randomness. Regarding the treatment of patients, there may have been a selection bias. PSM analysis could not eliminate all these biases. Second, more than 90% of patients with hepatocellular carcinoma have hepatitis B virus infection. As a result, these data may not apply to Western countries, where hepatocellular carcinoma is more often caused by alcohol use and HCV. Furthermore, this was a single-centre study, potentially limiting the generalizability of the results.

## Conclusion

Compared with hepatectomy alone, preoperative TACE before hepatectomy resulted in favourable treatment responses and improved long-term survival outcomes. Moreover, the survival of hepatectomy patients who achieved complete remission of tumours after TACE was significantly prolonged. Therefore, preoperative TACE may be an ideal therapeutic strategy for surgically treating HCC (single > 3 cm or multiple) to reduce recurrence.

## Data Availability

The original contributions presented in the study are included in the article/Supplementary Material, and further inquiries can be directed to the corresponding author.

## Declarations

### Synopsis for table of contents

The objective of this study was to compare the effects of preoperative transcatheter arterial chemoembolization (TACE) and non-TACE on long-term survival in patients who undergo radical hepatectomy.

## Abbreviations

AFP: Alpha-fetoprotein
ALP: Alkaline phosphatase
ALT: Alanine aminotransferase
AST: Aspartate aminotransferase
BCLC: Barcelona Clinical Hepatocellular Carcinoma
CR: Complete response
DSA: Digital subtraction angiography
HCC: Hepatocellular carcinoma
MRI: Magnetic resonance imaging
OS: Overall survival
PD: Progressive disease
PFS: Progression-free survival
PNI: Prognostic nutritional index
PR: Partial response
PSM: Propensity score matching
RFA: Radiofrequency ablation
SD: Standard deviation
SD: Stable disease
TACE: Transcatheter arterial chemoembolization
TBIL: Total bilirubin
